# One-stage laser-microtextured implants immediately placed in the inter-radicular septum of molar fresh extraction sockets associated with GBR technique. A case series study

**DOI:** 10.4317/jced.54705

**Published:** 2018-10-01

**Authors:** Renzo Guarnieri, Dario Di Nardo, Gianfranco Gaimari, Gabriele Miccoli, Luca Testarelli

**Affiliations:** 1 MD DDS, Adjunct Professor. Dept. of Dental and Maxillofacial Sciences, School of Dentistry, University La Sapienza, Rome, Italy; 2 DDS, PhD. Dept. of Dental and Maxillofacial Sciences, School of Dentistry, University La Sapienza, Rome, Italy; 3 DDS, PhD, Associated Professors. Dept. of Dental and Maxillofacial Sciences, School of Dentistry, University La Sapienza, Rome, Italy

## Abstract

**Background:**

The outcome of one-stage trans-mucosal immediate implants with simultaneous guided bone regeneration (GBR) technique has become highly predictable. Furthermore, when this approach is performed to place one-stage implants into the inter-radicular septum of fresh extraction sockets in the molar region, the risk of incorrect emergence profile and off-angle loading is reduced. The aim of the present study was to clinically evaluate the horizontal hard and soft tissue changes, and radiographically the vertical socket walls remodeling, and the early peri-implant marginal bone loss (EMBL) following the placement of immediate one-stage implants in the inter-radicular septum of molar fresh extraction sockets, associated with a collagen membrane.

**Material and Methods:**

Twenty patients were selected to receive a one-stage implant with laser-microtextured collar surface into the inter-radicular septum of a fresh molar extraction sockets, associated with a simultaneous placement of a collagen membrane. Intraoral radiographs and model casts were used for the evaluation. Correlation between the amount of the keratinized tissue thickness (KTT) with EMBL was also analyzed.

**Results:**

After 4 months, the vertical radiographic mesial and distal EMBL around implants was of 0.06 ±0.01 mm and 0.04±0.02 mm, respectively, with no statistically significant difference between T0 and T1 (*P* >0.05). No statistical differences were found also for each radiographic measure used for the examination of implant sites vertical bone changes (*p* >0.05). Clinically, horizontal changes of the bucco-lingual central width were found statistically significant (*p*<0.05), whereas no statistical differences were found for bucco-lingual mesial and distal width changes (*p* >0.05). In addition, no statistically significant correlation between EMBL and the amount of KTT was found (*P* >0.05).

**Conclusions:**

Results suggest that the immediate placement of one-stage laser-microtextured implants could provide advantages in preserving the extraction socket’s hard and soft tissue remodeling, and the peri-implant marginal bone level before the prosthetic loading.

** Key words:**One-stage implant, laser-microtextured collar surface, GBR, collagen membrane.

## Introduction

The need to reduce the number of surgical interventions and the implant treatment times, have led clinicians and researchers to develop a new dental implant placement protocol, defined with the term of “immediate implant placement” (IIP) (1). It provides the insertion of the implant immediately after extraction of the tooth to be substituted (1). Several advantages have been described for this protocol, such as reduction of the number of surgical procedure, preservation of alveolar bone volume, optimal soft tissue esthetics, ideal orientation of the implant, and increased patient comfort and satisfaction (1,2). In esthetic zones, the success rates of IIP replacing a single tooth are like those obtained with the delayed implant placement protocol into healed extraction sockets (1,2). Consequently, IIP has gained acceptance among clinicians particularly in the esthetic zones. On the contrary, because of presence of larger extraction sockets, poor quality of bone, and less apical bone availability (for the proximity of maxillary sinus and alveolar inferior nerve) clinicians commonly avoided immediate implant placement in molar extraction sockets (3). Despite the above-mentioned limitations, IIP protocol in molar fresh extraction sites could present some advantages if implant is placed into the inter-radicular septum. Simultaneous GBR with immediate implant placement was thought to be possible only in a submerged environment. However, a series of studies have suggested that high predictability of immediate implants with simultaneous GBR technique can also be achieved with a one-step trans-mucosal-healing approach (4-7). The aim of the present study was to clinically evaluate the horizontal hard and soft tissue changes, and, radiographically, the vertical socket walls remodeling and the early peri-implant marginal bone loss (EMBL), following the placement of immediate one-stage implants with laser-microtextured collar surface in the inter-radicular septum of molar fresh extraction sockets, associated with a collagen membrane.

## Material and Methods

Patient selection: Twenty patients, who required implant therapy for the replacement of mandibular or maxillary hopeless molar teeth, were identified and enrolled in this study. Criteria for inclusion were: age ≥ 18 years, good general health, presence of molar extraction socket type 1 according with the classification suggested by Juodzbalys *et al.* (8) ( Table 1), and a presence of inter radicular septum with a sufficient amount of bone to place a standard implant (3.8 mm diameter and 9 mm length), detectable by means of CBCT evaluation. Exclusion criteria were: natural teeth adjacent to surgical area affected by untreated periodontal or endodontic infections, absence of opposing occlusion, full-mouth plaque score (FMPS) ≥25%; full-mouth bleeding score (FMBS) ≥25% recorded at the time of implant placement, para-functional habits, severe maxilla-mandibular space discrepancies, uncontrolled diabetes, treatment with bisphosphonates, patients smoking >10 cigarettes a day, and any drug/alcohol abuse. All patients were informed about the evidence-based, positive outcome of the IIP approach associated with GBR technique that were tested. Each patient signed a free informed consent form after he/she has received detailed information about the study. Treatments were performed according to the principles outlined in the Declaration of Helsinki on experimentation involving human subjects.

**Table 1:**
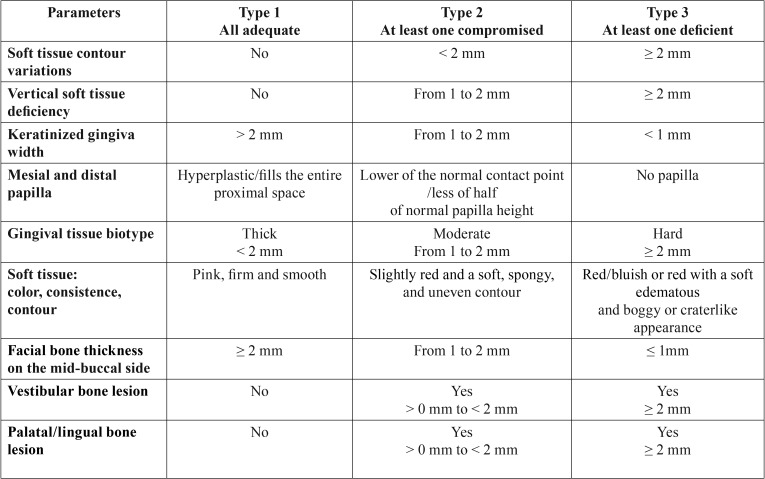
Extraction socket classification according to Juodzbalys, *et al.* (8).

Implants: Twenty BioHorizons Tissue Level Laser-Lok® Implants (Biohorizons, Birmingham; Al, USA) were used for the study. Implants have the body grit-blasted to create a moderately rough surface (roughness between 0.72 and 1.34µm), while the apical 2.0 mm of the collar are characterized by the presence of laser-produced microgrooves on the range of 8µm, and the most coronal 1.3mm of the collar is smooth, machined metal.

**Figure 1:**
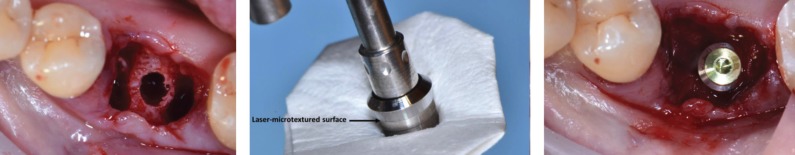
Preparation of the inter radicular recipient site (right); the membrane is cropped according to the measurements of the post-extraction socket perimeter, and the implant is inserted into the center of the membrane exactly in the transverse area between the surface of the implant body and the laser-microtextured collar (center); simultaneous placement of implant and membrane (right)).

Surgical procedure: All implants were placed by the same operators (RG, LT). All the subjects adopted an antimicrobial prophylaxis with mouthrinses of 0.12% chlorhexidine 1-minute rinse before surgery and three times a day for the following 10 days (Dentosan 0.12%, Johnson & Johnson, USA). Amoxicillin + clavulanic acid 1 g bid (Augmentin, Glaxo SmithKleine, Italy) was prescribed for 7 days. Local anesthesia was induced by infiltration with articaine/epinephrine (1:100.000) (Ecocain 20mg/ml, Molteni Dental, Italy). Each molar tooth, if necessary, was sectioned to make the extraction the least traumatic possible, and a flapless procedure was performed for the extraction. The preparation of the inter-radicular recipient site was performed following the instructions of implant manufacturer under abundant saline solution irrigation. A collagen membrane (Mem-Lok Pliable®, BioHorizons, Birmingham, AL, USA) was used in each molar extraction socket. Mem-Lok Pliable® is a porcine-derived resorbable collagen-based membrane with an estimated resorption time of 12-14 weeks. Before the positioning, the membrane was cropped according to the measurements of the post-extraction socket perimeter, and the implant was inserted into the center of the membrane exactly in the transverse area between the surface of the implant body and the laser-microtextured collar. In this way the simultaneous implant and membrane placement allows to position the laser-microtextured implant collar above the inter-radicular septum, and the membrane above the lingual/palatal and vestibular bone crest (Fig. 1). Before the simultaneous implant and membrane placement, the interdental mesial and distal papilla was prepared with a pouch procedure, to allow, mesially and distally, the placement of the membrane under the interdental papilla. In this way, the membrane is sustained in the center by the implant, and along its perimeter by the extraction socket walls, leaving the laser-microtextured collar in contact with the soft tissue. Sutures were used to stabilize the membrane, which was left exposed to the oral cavity. Patients were instructed to have a liquid or semiliquid diet for the first three days and then gradually return to a normal diet. An analgesic (Ibuprofen®, 600 mg) immediately after the surgical intervention and after 8 hours were prescribed.

**Figure 2:**
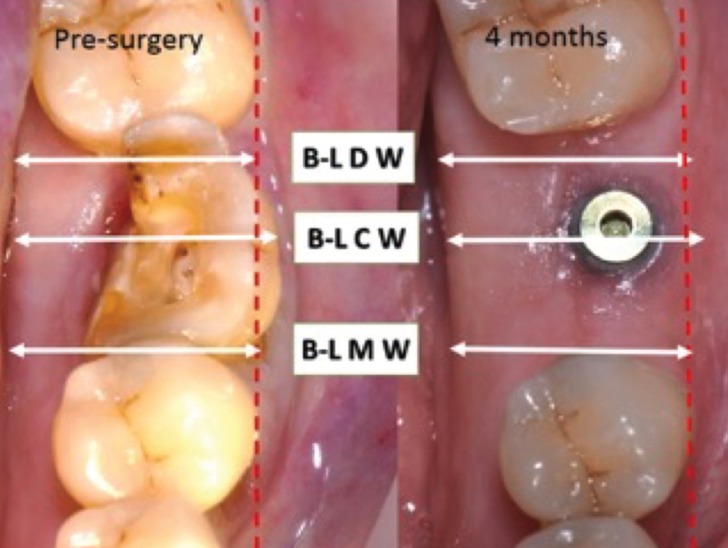
Horizontal tissue changes of the implant site before the tooth extraction and after 4 months.

Clinical examination: before tooth extraction, and 4 months following the implant placement, model casts were used to clinically measure the horizontal tissue changes of the implant site. The following clinical measurements were performed (Fig. 2).

• B-L DW = bucco-lingual distal width (mm), 

• B-L CW = bucco-lingual central width (mm), 

• B-L MW = bucco-lingual mesial width (mm).

In cast models, each horizontal measure was performed with a caliber among the distal, central, and mesial higher point of alveolar buccal and lingual edge. The correlation between the amount of the keratinized tissue thickness (KTT) with EMBL was also analyzed. Keratinized tissue thickness (KTT) was recorded before the extractive procedure. The KTT was measured in the vestibular aspect of the molar tooth by means of n. 30 K-file inserted until touching the bone crest. The KTT was dichotomized into two groups (≤2 mm and >2 mm) in accordance with the results of an animal study performed by Berglundh et al. (9), which was the first attempt to analyze the influence of mucosal thickness on peri-implant marginal bone loss.

**Figure 3:**
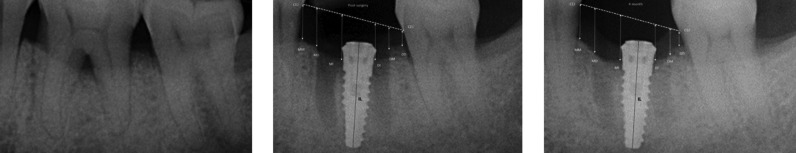
Rx taken before tooth extraction (left), at the immediate implant placement (center), and 4 months later (right).

Radiographic examination: Radiographs were performed immediately at the implant placement, and 4 months after surgery (T0 and T1, respectively) with a paralleling technique using a Rinn film holder with a rigid film-object X-ray source. For the radiograph procedures, a silicone index material was fixated to the residual dentition and a radiograph holder was constructed for each patient. This technique ensured that the same position of the radiograph film could be reproduced at each visit and the angle of the radiograph would not deviate. The radiographs were taken in high resolution mode (Vista Scan Durr Dental, Durr Dental Italy S.r.l) with a dental x-ray machine (TM 2002 Planmeca Proline CC, Planmeca Group Helsinki, Finland) equipped with a long tube that operated at 70 Kw/7.5 mA. Specialized software (DBSWIN software, Durr Dental Italy S.r.l) was used for linear measurements of marginal bone changes. The following radiographic measurements were performed (Fig. 3):

- radiographic implant length (IL): distance (in mm) between the implant shoulder and the implant apex as assessed at the mid portion of the implant;

- residual bone height at the mesial (MI) and distal (DI) aspects of the implant: distance (in mm) between the line linking the CEJs of the adjacent teeth, and the first contact of the crestal bone on both mesial and distal side of the implant.

- bone height at the mesial (MM) and distal (MD) aspects of the residual mesial extraction socket bone peak, and bone height at the mesial (DM) and distal (DD) aspects of the residual distal extraction socket bone peak, measured in mm from the line linking the CEJs of the adjacent teeth.

To account for radiographic distortion, radiographic measurements (i.e. MI, DI, MM, MD, DM, and DD) on each radiograph were adjusted for a coefficient derived from the ratio: true length of the implant/IL. For each implant, the EMBL was calculated as the mean value of MI and DI. All measurements were carried out by a single trained examiner who had previously undergone a calibration session for radiographic assessment on a sample of 10 patients treated with the same implant system and not included in the study (Kappa Test= 0.940, SE of kappa = 0.042, 95% confidence interval: from 0.857 to 1.000).

Statistical analysis: statistical analysis was performed using 13.0 SPSS® statistical program (SPSS, Chicago, IL, USA). Results were expressed as mean, standard deviation, median, and range. Data were analyzed by means of Mann–Whitney test. A *P* value < 0.05 was considered statistically significant.

## Results

After 4 months from the implant placement, the vertical radiographic mesial and distal EMBL around implants was of 0.06 ±0.01 mm and 0.04±0.02 mm, respectively, with no statistically significant difference between T0 and T1 (*P* > 0.05). No statistical differences were found also for each radiographic measure (MI, DI, MM, MD, DM, and DD) used for the examination of implant sites vertical bone changes (*p*>0.05) (Fig. 3,  Table 2). Clinically, the mean value of the B-L CW at T0 and T4 was 12.4mm (SD 1.8), and 10.9 mm (SD 1.2). The difference was found statistically significant (*p*<0.05). At T0, mean values of B-L MW and B-L DW were 9.8mm (SD 1.3) and 10.9 mm (SD 1.1), respectively, whereas at T1 mean value of B-L DW was 9.4 mm (SD 1.2) , and the mean value of B-L DW was 10.2 mm (SD 1.6) (Fig. 2). Differences were not statistically significant (*p*>0.05), ( Table 3). No statistically significant correlation was found also between EMBL and the amount of KTT (*P* > 0.05) ( Table 4).

**Table 2:**
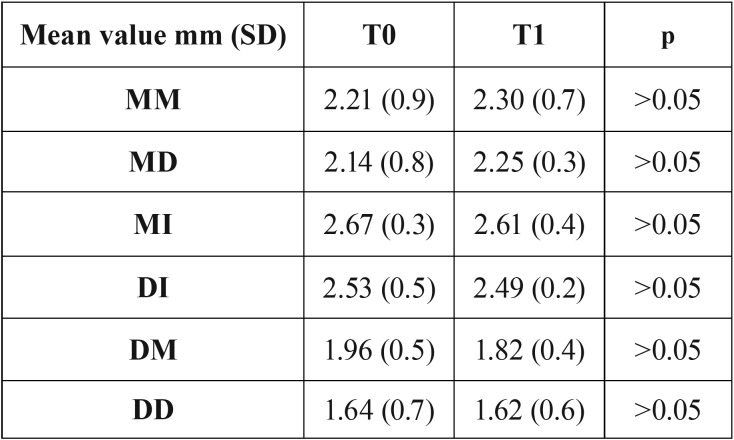
Radiographic measurements at the immediate implant placement (T0) and after 4 months (T1).

**Table 3:**
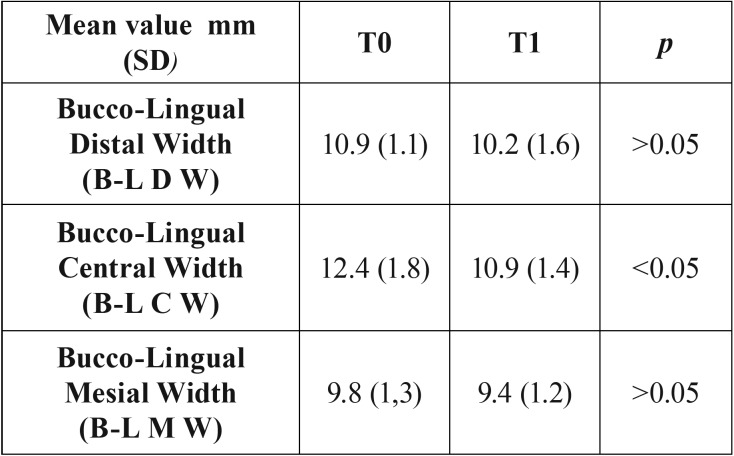
Clinical horizontal measurements before the immediate implant placement (T0) and after 4 months (T1).

**Table 4:**
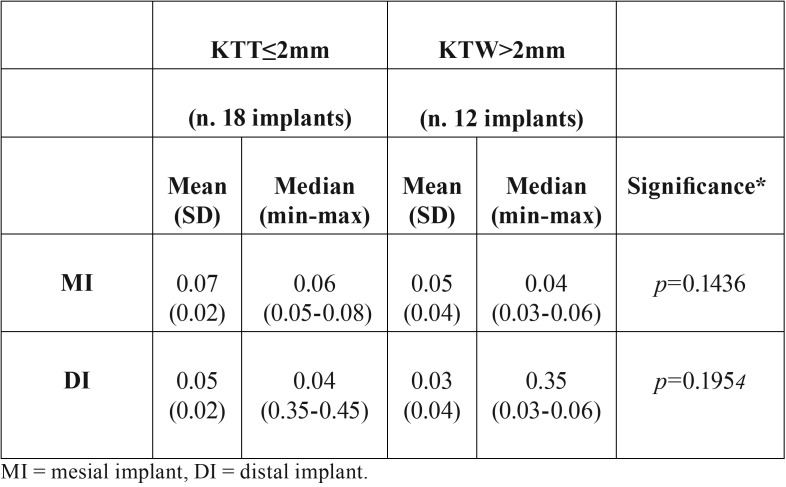
Mean EMBR value according with the KTT.

## Discussion

After tooth extraction, the alveolar bone walls reabsorption is slightly greater in extraction sockets of multi-rooted teeth than in single-rooted teeth (10-12). Accordingly, in cases of extraction of molar teeth, the primary treatment option should be aimed preventing the alveolar bone remodeling. Several studies indicate that immediate implant placement per se does not prevent reabsorption of the alveolar bone walls, and that the reabsorption of the buccal and lingual walls at 3 months is similar compared with extraction only sites (13-16). However, in many of reported studies no attempt was made at the time of implant placement to treat the peri-implant defects by applying the principles of Guided Bone Regeneration (GBR). On the contrary, in other studies, where a GBR technique associated with the immediate implant placement was used, marginal defects around immediate implants in extraction molar sockets were found filled after 6 months of healing (17,18). Literature data indicated that the success and survival rate of immediate implantation into fresh extraction socket of maxillary molars is more than 90%, which is like conventional type delayed implants (3). Accordingly, immediate implant placement in maxillary molar extraction sockets appear to be a predictable procedure if proper case selection is conducted. Careful case selection is due to a number of risk factors that may jeopardize IIP treatment in fresh extraction sockets of posterior mandible and maxilla, such as the difficulty in achieving primary implant stability, the higher occlusal forces, and the limited bone quantity caused by the presence of adjacent vital structures such as the mandibular nerve and maxillary sinus (19,20). Wide-diameter implants (>4.5mm) have been proposed to overcome these limitations. However, reported clinical outcomes of wide-diameter implants are contradictory. Some studies showed a higher failure rate for wide-diameter implants compared with the standard size implants (21,22), while some other studies indicated high success rates (23,24). Differences may be related to implant design and surface; thus it is difficult drawing conclusions. Molar extraction sockets, especially in mandibular jaw, present a wider mesiodistal than bucco-lingual space, consequently the maximum contact between a wide-diameter implant and alveolar bone is located at the vestibular and lingual wall. The anatomical discrepancy between a wide diameter implant and the form of molar extraction socket may not allow an equal stress distribution, contributing to the higher stress concentration in the buccal cortical bone. The high compressive stress at the dense marginal bone in the posterior mandible may result in microcracks and bone resorption, which may subsequently lead to early implant failure (25). An alternative in achieving primary stability in a fresh molar extraction socket is the placement of the implant exactly in the inter-radicular septum. The placement of an implant in the inter-radicular septum also allows to reduce the risk of incorrect emergency profile, off angle loading and the creation of a cantilever (26). A tapered self-tapping implant placed in the inter-radicular septum could offer similar potential advantages to achieve primary implant stability of a wide-diameter implant without the over-mentioned risks (27).

The EMBL that occurs after implant placement is related to the biologic width associated with implants (28). Laser-microtextured collar implant allows a perpendicular/functional organization of connective tissue fibers, that counteracts the downgrouth of epithelium, limiting the EMBL, both in native bone, and in post-extractive fresh sites (29,30). An histological study in a dog by Shin & Han (30), compared the alveolar bone reduction after immediate implantation of microgrooved and smooth collar implants in fresh extracted sockets. Results documented in the supracrestal region variations in the attachment and orientation of the connective tissue along the surface texture of implants. In the microgrooved group, the collagen fibers showed a perpendicular orientation to the implant over the 8µm pitch microgrooved surface, whereas in the turned surface group, the fibers were parallel to the fixtures. In addition, in the microgroove groups, the epithelium migrated down to where the connective tissue was attached, whereas in the turned surface group, the epithelium grew downward to where the thread began passing over the turned surface. The bone-implant marginal gaps in both groups were less than 1.5 mm, and no regenerative technique was used in association with the implant placement. Contrarily, in molar extraction sockets treated in the present study, the mesial and distal residual alveolar bone defect usually was >2mm, and a regenerative technique was needed. The GBR technique proposed allows at the same time leaving free the laser-microtextured collar above the reasorbable membrane totally in contact with the soft tissue of post-extractive site during the healing phase, while the membrane covers the extraction socket’s mesial and distal alveolar bone defects. In the present study, histological evaluations have not been carried out, however, clinical and radiographic outcomes suggested that the laser-microtextured surface with 8µm pitch allows to obtain a stable soft tissue seal around implant collar, protecting the peri-implant marginal bone, also using an trans-mucosal-healing approach associated with a GBR technique.

Some limitations of the present study must be highlighted. The absence of histological data (that has been already mentioned), and the absence of a control group of implants without laser-microtextured collar, require further studies to confirm our findings. However, preliminary results of clinical studies such as this one often represent the first line of clinical evidence, which underscores their clinical value.

## Conclusions

Within the limits of the present study, it is possible conclude that one-stage Tissue Level Laser-Lok® implants, immediately placed in the inter-radicular septum of molar fresh extraction sockets, associated with a GBR technique, could provide advantages in preserving extraction socket’s bone remodeling and the peri-implant marginal bone level before the prosthetic loading.

Clinical significance, Since it has been histologically documented in humans a perpendicular/functional organization of connective tissue fibers around a laser-microtextured collar implant, the GBR technique proposed in the present study allows the laser-microtextured collar to be positioned above the reabsorbable membrane, totally in contact with the soft tissue during the healing phase, with the membrane covering the extraction socket’s mesial and distal alveolar bone defects, and marginal defects around one-stage implants. Furthermore, using this approach the placement of a one-stage implant into the inter-radicular septum of fresh extraction sockets in the molar region, reduces the risk of incorrect emergence profile and off-angle loading..

Conflicts of interest: The authors report no conflicts of interest related to this study. Authors have no financial and personal relationships with other people or organizations that could inappropriately influence their work in case. Materials to the study were provided by BioHorizons, Birmingham, AL; USA.
